# Correction: Mating-induced neurogenesis and cell proliferation in male rats depend on opioid signaling

**DOI:** 10.1371/journal.pone.0341296

**Published:** 2026-01-20

**Authors:** The PLOS One Staff

## Notice of Republication

This article was republished on January 5, 2025, to correct Figures 7, 8, and 9

## Supporting information

S1 File Originally published, uncorrected article. (PDF)

S2 File Republished, corrected article. (PDF)
