# Supplementary material for: Correction: Mating-induced neurogenesis and cell proliferation in male rats depend on opioid signaling
Source: PLoS One. 2026 Jan 20;21(1):e0341296. doi: 10.1371/journal.pone.0341296 (PMC12818631; doi:10.1371/journal.pone.0341296)
Supplement: S2 File — (PDF) [file pone.0341296.s002.pdf]

RESEARCH ARTICLE

Mating-induced neurogenesis and cell proliferation in male rats depend on opioid signaling

Marie Bedos[1]*, **Mariana Sánchez Núñez**[2], **Analía E. Castro**[1,2], **Raúl G. Paredes**[1,2]

1 Escuela Nacional de Estudios Superiores Unidad Juriquilla, Campus UNAM-Juriquilla, Universidad Nacional Autónoma de México, Querétaro, Querétaro, Mexico, **2** Instituto de Neurobiología, Campus UNAM-Juriquilla, Universidad Nacional Autónoma de México, Querétaro, Querétaro, Mexico

* bedos@unam.mx

Abstract

In the adult brain, neurogenesis primarily occurs in the dentate gyrus of the hippocampus (DG) and the olfactory bulbs, with new cells migrating from the subventricular zone. Additionally, small amounts of cell proliferation have been observed in the preoptic area (POA) and the amygdala (AMG), regions involved in the control of male sexual behavior. Sexual activity induces a reward state mediated by opioids, and our group previously demonstrated that neurogenesis induced by paced mating is opioid dependent in female rats. Therefore, in the present study, we examined whether naloxone hydrochloride could block the cell proliferation and neurogenesis induced by mating in male rats. We evaluated cell proliferation and neurogenesis in the DG, main (MOB) and accessory (AOB) olfactory bulbs, POA and AMG across 6 groups of male rats: without sexual contact injected with saline or NX, males that mated until they ejaculated once injected with saline or NX and males that mated until they ejaculated 3 times injected with saline or NX. Our findings indicated that the increase of cell proliferation and neurogenesis observed after 3 ejaculations was abolished by NX administration in the glomerular layer of both the AOB and MOB. The same effect was observed in the granular layer of the MOB. In contrast, NX did not reduce the cell proliferation induced by 3 ejaculations in the granular layer of the AOB, but significantly reduced neurogenesis. In the DG, cell proliferation and neurogenesis were increased by 3 ejaculations and NX blocked this effect. Finally, analyses of the AMG and POA revealed that NX blocked the cell proliferation induced by 3 ejaculations. This study highlights the central role of opioid signaling in mediating the effects of sexual behavior on cell proliferation and neurogenesis in both classical and non-classical neurogenic regions.

OPEN ACCESS

Citation: Bedos M, Sánchez Núñez M, Castro AE, Paredes RG (2025) Mating-induced neurogenesis and cell proliferation in male rats depend on opioid signaling. PLoS One 20(9): e0329795. https://doi.org/10.1371/journal.pone.0329795

Editor: Juan M Dominguez, University of Texas at Austin, UNITED STATES OF AMERICA

Received: April 8, 2025

Accepted: July 21, 2025

Published: September 4, 2025

Copyright: © 2025 Bedos et al. This is an open access article distributed under the terms of the Creative Commons Attribution License, which permits unrestricted use, distribution, and reproduction in any medium, provided the original author and source are credited.

Data availability statement: All relevant data are within the paper and its Supporting information files.

Funding: This research was funded by Dirección General de Asuntos del Personal Académico (DGAPA, https://dgapa.unam.mx/) of the Universidad Nacional Autónoma

de México (UNAM), through grants IN214524 (RGP) and IA208624 (MB) of the Programa de Apoyo a Proyectos de Investigación e Innovación Tecnológica (PAPIIT). The funders did not play any role in the study design, data collection and analysis, decision to publish, or preparation of the manuscript.

Competing interests: The authors have declared that no competing interests exist.

Introduction

It is well established that neurogenesis, one of the plastic processes that occurs in adult brains, takes place in 2 principal regions of mammalian brains: the dentate gyrus of the hippocampus and the subventricular zone of the lateral ventricles [1,2]. New cells proliferate from the latter and travel through the rostral migratory stream to reach the olfactory bulbs and eventually integrate into circuits [3]. In the hippocampus, new cells divide in the subgranular zone and integrate nearby circuits [4,5]. This neuroplastic phenomenon is modulated by a variety of endogenous and exogenous factors such as hormones, trophic factors, neurotransmitters, and sensorial stimuli, and their actions can impact one or several stages of the process, namely proliferation, migration, incorporation, and survival [6,7].

Sexual behavior is an important component of the reproductive function, and the structures involved in its control are well studied. In both sexes, the possibility of controlling the rate of the sexual interaction (pacing) induces a positive affective state, as tested with the conditioned place preference paradigm [8–11]. Interestingly, we found that the systemic administration of naloxone hydrochloride, an opioid antagonist, blocked the reward state induced after sexual behavior, as females did not change their original preference, suggesting that opioids are involved in the positive affective state produced by paced mating [12]. In particular, infusions of naloxone methiodide into the medial preoptic area, ventromedial nucleus of the hypothalamus, and amygdala blocked conditioned place preference induced by paced mating behavior in females [13]. Similarly, the systemic administration of naloxone hydrochloride in males blocked the reward state induced by paced mating [14,15]. In particular, methylnaloxonium infused in the medial preoptic area of male rats blocked the reward state produced by mating [16]. However, naloxone hydrochloride administration did not affect sexual behavior in males [17] or females [18].

Sexual behavior in males increased the number of new cells that incorporate into the rostral migratory stream and the granular layer of the olfactory bulb. As well, sexual experience increased cell proliferation in both females and males. Mating also restored proliferation rates in middle-aged rats [19,20].

Several neurotransmitters involved in social behaviors, including oxytocin [21] and opioids [22], also contribute to the regulation of cell proliferation and neurogenesis in the adult brain. Interestingly, we found that the increase in the number of new cells observed in the olfactory bulb after paced mating is blocked by i.p. injection of naloxone hydrochloride [18]. These results suggest that endogenous opioids participate in the increase of neurogenesis induced by paced mating in female rats.

Therefore, in the present study, we evaluated if the systemic injection of naloxone hydrochloride in males allowed to copulate until ejaculation would decrease the number of new cells in the olfactory bulb and the dentate gyrus of the hippocampus. We hypothesized that the number of new cells that differentiated into neurons would decrease in the presence of the opioid antagonist. Since small amounts of cell proliferation occur in the preoptic area and amygdala [23,24], and both regions play a role in the control of male sexual behavior, we included them in the analysis.

Materials and methods

Subjects

Male Wistar rats (250–350 g) were obtained from the local colony at the animal facility of the Instituto de Neurobiología, UNAM. They were allowed to copulate during 3 pre-test mating sessions (until ejaculation or a maximum of 1h) to give them sexual experience. Males were housed in groups of 3–4 in acrylic cages (18.5 × 20 × 10 cm) covered with fresh sawdust. They were maintained in a room with controlled temperature (25 ± 1°C) under an inversed 12h:12h light–dark cycle, starting the dark phase at 08:00. Animals were fed with standard laboratory rat chow and water ad libitum. Stimulus females (200–250 g) of the same strain were bilaterally ovariectomized under general anesthesia. Receptivity was induced by the subcutaneous administration of estradiol benzoate (25 µg/rat) and progesterone (1 mg/rat), 48 h and 4 h, respectively, before mating tests. The steroids were dissolved in corn oil and injected in a volume of 0.2 ml/rat. This study was carried out in accordance with the recommendations of Reglamento de la Ley General de Salud en Materia de Investigación para la Salud, NOM-062-ZOO-1999 of the Mexican Health Ministry, ~~that~~ which follows NIH guidelines. The protocol 097-A was approved by the Bioethics Committee of the Institute of Neurobiology.

Behavioral tests

Sexual behavior tests were performed at 12:00 h in acrylic cages (40 × 60 × 40 cm) with clean sawdust under dim red light. Subjects were randomly assigned to one of six groups: 1) Males without sexual contact injected with saline (Control Saline); 2) Males without sexual contact injected with naloxone hydrochloride (Control NX); 3) Males injected with saline that mated until they ejaculated once (1 EJ saline); 4) Males injected with naloxone hydrochloride that mated until they ejaculated once (1 EJ NX); 5) Males injected with saline that mated until they ejaculated 3 times (3 EJ saline) and 6) Males injected with naloxone hydrochloride that mated until they ejaculated 3 times (3 EJ NX). In control groups, males were placed alone in the sexual behavior cages for 60 min. Animals that received the opioid antagonist were injected i.p. with naloxone hydrochloride (4 mg/kg in 1 ml of saline; Du Pont, México). The behavioral tests lasted until males had reached their first or third ejaculation, depending on the group.

BrdU administration

Males were injected i.p. with the DNA synthesis marker 5'bromo2'deoxyuridine (BrdU, Sigma-Aldrich) to assess cell proliferation. They received 3 injections of 100 mg/kg each: 1h before the behavioral test, at the end of the test, and 1h after the test ended.

Tissue preparation

After the behavioral test, males were returned to their home cages, and 15 days later, they were euthanized by i.p. injection of pentobarbital (60 mg/kg, Cheminova). Animals were intracardially perfused with phosphate-buffered saline (PBS, 0.1 M and pH 7.2 J.T. Baker) and paraformaldehyde (4%, Sigma). Brains were removed and stored at 4 °C in 30% sucrose (J.T. Baker) dissolved in PBS.

Brains were sliced in a cryostat (Leica CM 1850). Sagittal sections (30-µm) containing the MOB and AOB were collected in 3 series. The first series was processed for immunohistochemistry and revealed by diaminobenzidine to visualize the new cells (BrdU-positive). The second series was processed for immunofluorescence to determine if the new cells differentiated into neurons (NeuN, neuron-specific nuclear protein). We randomly selected 7 and 4 subjects for immunohistochemistry and immunofluorescence, respectively.

Immunohistochemistry for BrdU

Free-floating sections were washed 4 times in Tris-buffered saline (TBS 0.1 M, pH 7.6 J.T Baker) and incubated for 30 min in Triton X-100 (0.1%, J.T Baker) and 30% H_2O_2 (1%, J.T Baker) to eliminate endogenous peroxidase activity. Tissue was rinsed

in TBS and incubated in 2N HCL (J.T Baker) at 37°C for 60 min. The sections were washed in TBS and incubated for 30 min in bovine albumin (1%, Sigma-Aldrich) and Triton X-100 (0.3%) to block unspecific epitopes. The tissue was incubated for at least 16 h at 4°C in primary antibody, mouse monoclonal anti-BrdU (BD Biosciences, dilution 1:2000) in 0.1% bovine albumin. The sections were rinsed in TBS containing Triton X-100 (0.02%) and bovine albumin (1%) and then incubated with biotinylated anti-mouse IgG (1:500, Vector Laboratories) for 2h. The sections were rinsed in TBS containing Triton X-100 (0.02%) and incubated at RT in Avidin Biotin Complex (ABC elite kit, Vector Laboratories) for 90 min. Tissue was then washed in TBS and revealed with nickel chloride-3,3'-diaminobenzidine (DAB, Vector Laboratories) and H_2O_2. The reaction was stopped by washing the sections in TBS. Finally, the brain sections were mounted on gelatin-coated slides and cover slipped using Permount (Fisher Scientific).

Double-immunofluorescence assay for BrdU and NeuN

Double-immunolabeling protocol for detecting new proliferating neurons in the OB and DG was modified and adapted from previously published procedures [18,25]. After 4 washes for 5 minutes each in Tris-buffered saline (TBS 0.1 M, pH 7.6 J.T Baker), free-floating sections were incubated for 30 min in 3% H_2O_2-TBS (J.T Baker) to eliminate endogenous peroxidase activity. Tissue was rinsed in TBS, incubated in 0.1 M citrate buffer (Sigma-Aldrich) pH 6 for 30 min and subsequently incubated in 2N HCl (J.T Baker) at 50°C for 60 min. The sections were washed 3 times for 5 minutes each in TBS and incubated for 30 min in bovine albumin BSA (0.5% w/v bovine serum albumin fraction V A9418 (Sigma-Aldrich) in Triton X-100 (0.3%) to block unspecific epitopes. The tissue was incubated for 1 h at room temperature (RT) and then, at least 16 h at 4°C in primary antibody, rat anti-BrdU (ab6326 Abcam, dilution 1:1 500) in 0.1% BSA-TBS. The sections were rinsed in TBS containing Triton X-100 (0.02%) and then incubated with biotinylated anti rat IgG (dilution 1:2000, BA-9400 Vector Laboratories) for 1h at RT. The sections were rinsed in TBS containing Triton X-100 (0.02%) 2 times for 5 minutes each and incubated at RT in Avidin Biotin Complex (ABC elite kit PK-6100, Vector Laboratories) for 1 h. Then, slides were rinsed 2 times for 5 minutes each and incubated with Cy3-Tyramide complex (TSA Plus Cyanine 3 System NEL744001KT), diluted 1:100 in the kit-specific diluent for 10 min in a wet chamber and washed 3 times for 5 min each with 0.02% TBS-Triton X100. A second blocking step using 0.5% BSA-TBS for 30 min at RT was performed and then, slides were incubated with a mouse anti-NeuN primary antibody (MAB377 Millipore, dilution 1:250) diluted in 0.1% BSA-TBS solution 1h at RT and overnight at 4 °C. Thus, slides were washed 3 times for 5 minutes each in 0.02% TBS-Triton X100 and incubated with an anti-mouse Alexa 488 antibody (A11001 Invitrogen, dilution 1: 1000) 1.5h at RT. Finally, sections were rinsed in 0.02% TBS-Triton X100 twice, mounted, covered with Aqua-Poly/Mount (Polysciences), and stored at 4°C in darkness until image acquisition.

Quantitative analysis of BrdU labeling

BrdU-DAB-stained tissue images were obtained with a light microscope, OLYMPUS BX60F-3 (10X objective), connected to a motorized slide (Prior ProScan) and analyzed using Image J software. BrdU-IR cells were identified and quantified in sagittal slices. The glomerular, mitral, and granular cell layers of the MOB and AOB were analyzed. Cell counting was performed according to previously reported studies in the OB [18]. The region of interest (ROI) in both the MOB and AOB was delimited with three circles in each layer. The diameters of the circles used in the MOB and AOB were 400 μm and 200 μm, respectively, as previously described [26]. The total ROI per layer was calculated with the sum of areas in each circle. The number of new cells was obtained with the average number of BrdU-IR positive cells in the total ROI per layer. In the dentate gyrus of the hippocampus, medial preoptic area, and amygdala, ROIs were freehand delimited using ImageJ software (Fig 1), and counts were reported as the average number of BrdU-IR positive cells per mm^2. An average of 3 sections per area per animal were quantified (n = 8 or 7 subjects per group).

Quantitative analysis of BrdU/NeuN-IR cells

Cell counting of BrdU/NeuN double-immunolabeling from MOB, AOB, and DG was carried out according to previously reported methodology [25]. Three sections per area per animal were captured using a confocal microscope Zeiss AX10

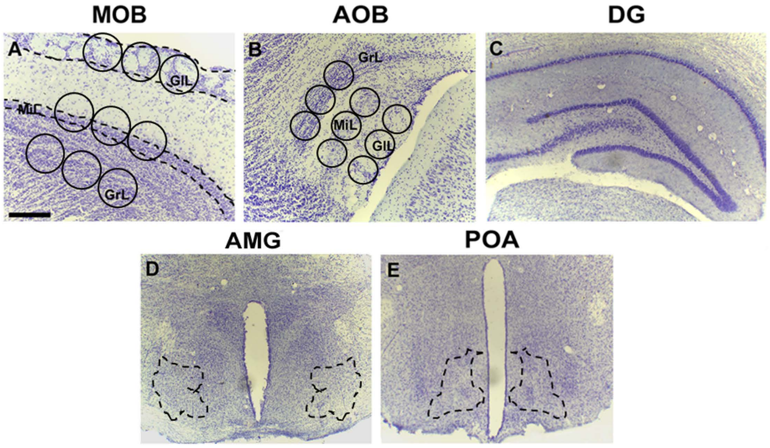

Fig 1. Representative images of Nissl staining for the rat brain regions analyzed in this study. (A-B) The olfactory bulbs were divided into the main (MOB) and accessory (AOB) subregions. For cell counting analysis, both OB regions were further subdivided into the classical olfactory cell layers: the glomerular layer (GlL), the mitral layer (Mi), and the granular layer (GrL). Circles indicate the regions of interest (ROIs) used for IR-cell quantification. ROI diameters: 400 μm in the MOB and 200 μm in the AOB. (C) Dentate gyrus of the hippocampus (DG), (D) amygdala (AMG), and (E) preoptic area (POA). Dashed lines delineate the areas selected for cell quantification. Scale bar: 200 μm.

https://doi.org/10.1371/journal.pone.0329795.g001

with a 20x objective and later were analyzed with ImageJ Software. MOB and AOB were subdivided into the glomerular cell layer (GrL) and granular cell layer (GrL) for IR cells quantification. First, images were separated into red and green channels to individually analyze BrdU and NeuN labeling. Size (50-infinity) and circularity (0.50–1.00) filters were applied using ImageJ software to exclude unspecific particles. Nuclei particles that co-localized for both specific IR-labeling were considered and quantified as double-IR cells. The regions of interest (ROIs) were delimited as described in the BrdU-IR analysis. The average number of BrdU/NeuN-IR cells obtained per area was extrapolated to IR- cells/mm^2.

Statistical analysis

All statistical analyses were performed using R (version 4.3.1). All datasets (S1 File) were tested for normality with the Shapiro-Wilk test. Since the data of most groups for cell proliferation, neurogenesis and sexual behavior were not normally distributed, we used a Kruskal-Wallis test to evaluate the overall differences between groups. In the case of cell proliferation and neurogenesis, the data were analyzed separately for each cell layer of the MOB and AOB. When appropriate, Kruskal-Wallis tests were followed by Dunn's multiple-comparisons post hoc test. Cell proliferation and neurogenesis data are presented as box plots with individual distribution. Behavioral data is presented as mean ± standard error. Group difference was considered significant at $p < 0.05$.

Results

Sexual behavior

Kruskal-Wallis tests revealed differences between groups for both mount and intromission numbers ($H(3)= 21.07$, $p < 0.01$) and $H(3)=24.46$, $p < 0.0001$, respectively; Table 1). Groups of males that were allowed to copulate until 3 ejaculations injected with saline or naloxone hydrochloride performed more mounts ($U = 4.18$, $p < 0.001$ and $U = 3.52$, $p < 0.01$, respectively) and intromissions ($U = 4.61$, $p < 0.0001$ and $U = 3.37$, $p < 0.01$, respectively) than males that ejaculated once and were injected with naloxone hydrochloride. Moreover, the 3 ejaculations + saline group displayed more intromissions than the 1 ejaculation + saline group ($U = 3.01$, $p < 0.05$). Additionally, the 3 ejaculations + saline group showed shorter III compared to males that ejaculated once and were injected with saline or naloxone hydrochloride ($U = 3.01$, $p < 0.05$ and

Table 1. Sexual behavior parameters of experimental males.

Group	1EJ Saline	1EJ NX	3EJ Saline	3EJ NX
**Latencies**				
Mount	101±33	143±61	45±9	70±28
Intromission	82±26	81±25	58±8	100±45
Ejaculation	885±128	1227±298	1533±625	683±69
**Number**				
Mount	15±2	9±0[a]	30±3[b]	25±3[b]
Intromission	16±1[c]	9±0[a,c]	37±2[b]	30±2[b,c]
PEI	1301±146	1652±304	1271±148	1064±68
III	65±8[c]	128±31[a,c]	101±49[b]	46±9[b,c]

Parameters of sexual behavior in males injected with saline that mated until a single ejaculation (1 EJ saline), males injected with naloxone hydrochloride that mated until a single ejaculation (1 EJ NX), males injected with saline that mated until three ejaculations (3 EJ saline), and males injected with naloxone hydrochloride that mated until three ejaculations (3 EJ NX). PEI: post-ejaculatory interval; III: inter-intromission interval. Columns labeled with different letters are significantly different from each other at $p < 0.05$.

https://doi.org/10.1371/journal.pone.0329795.t001

$U = 4.61$, $p < 0.0001$, respectively). The males that were allowed to copulate until 3 ejaculations and were injected with naloxone hydrochloride also showed shorter III than males that ejaculated once and were injected with naloxone hydrochloride ($U = 3.37$, $p < 0.01$).

Effect of naloxone hydrochloride and number of ejaculations on cell proliferation

Kruskal-Wallis tests revealed differences between groups in each layer of the MOB (glomerular: $H(5) = 17.95$, $p < 0.01$; mitral: $H(5) = 28.57$, $p < 0.0001$; granular: $H(5) = 26.67$, $p < 0.0001$). In all layers, the 3 ejaculations + saline group had more BrdU-IR cells than the control + NX group (glomerular: $U = -3.19$, $p < 0.05$, mitral: $U = -4.03$, $p < 0.001$, granular: $U = -4.38$, $p < 0.001$). Moreover, in both glomerular and mitral layers, 3 EJ saline induced more cell proliferation than 1 EJ NX ($U = 3.26$, $p < 0.05$ and $U = 3.66$, $p < 0.01$, respectively). Finally, in both the mitral and granular layers of the MOB, 3 ejaculations induced more cell proliferation than 1 ejaculation in males that were injected with saline ($U = 4.55$, $p < 0.0001$ and $U = 4.22$, $p < 0.001$, respectively). The groups treated with NX did not show a significant increase in cell proliferation in any of the layers of the MOB (Fig 2).

In the AOB, Kruskal-Wallis tests revealed differences between groups in each layer (glomerular: $H(5) = 25.29$, $p < 0.01$; mitral: $H(5) = 19.35$, $p < 0.01$; granular: $H(5) = 31.43$, $p < 0.0001$). In the glomerular and granular layers of the AOB, the 3 EJ saline group had more BrdU-IR cells than the control saline ($U = -3.3$, $p < 0.05$ and $U = -4.15$, $p < 0.001$, respectively) and control NX ($U = -3.84$, $p < 0.01$ and $U = -4.2$, $p < 0.001$, respectively). Furthermore, in the glomerular and mitral layers, 3 EJ saline induced more cell proliferation than 1 ejaculation NX ($U = 3.48$, $p < 0.01$ and $U = 3.45$, $p < 0.01$, respectively). In the mitral layer, 3 ejaculations induced more cell proliferation than 1 ejaculation in males that were injected with naloxone hydrochloride ($U = 3.17$, $p < 0.05$) and, in the granular layer, the 3 EJ NX group had more BrdU-IR cells than control groups, independently if they were injected with saline or NX ($U = -3.49$, $p < 0.01$ and $U = -3.54$, $p < 0.01$, respectively). Finally, in the glomerular layer, the 1 EJ saline group had more BrdU-IR cells than the Control NX group ($U = -2.9$, $p < 0.05$). In the mitral layer, the injection of naloxone hydrochloride decreased cell proliferation compared to males that received saline in males that ejaculated once ($U = 3.02$, $p < 0.05$; Fig 3).

In the hippocampus, Kruskal-Wallis test revealed differences between groups ($H(5) = 29.59$, $p < 0.0001$). The 3 EJ saline group had more BrdU-IR cells than the control groups, independently if they were injected with saline or NX ($U = -3.29$,

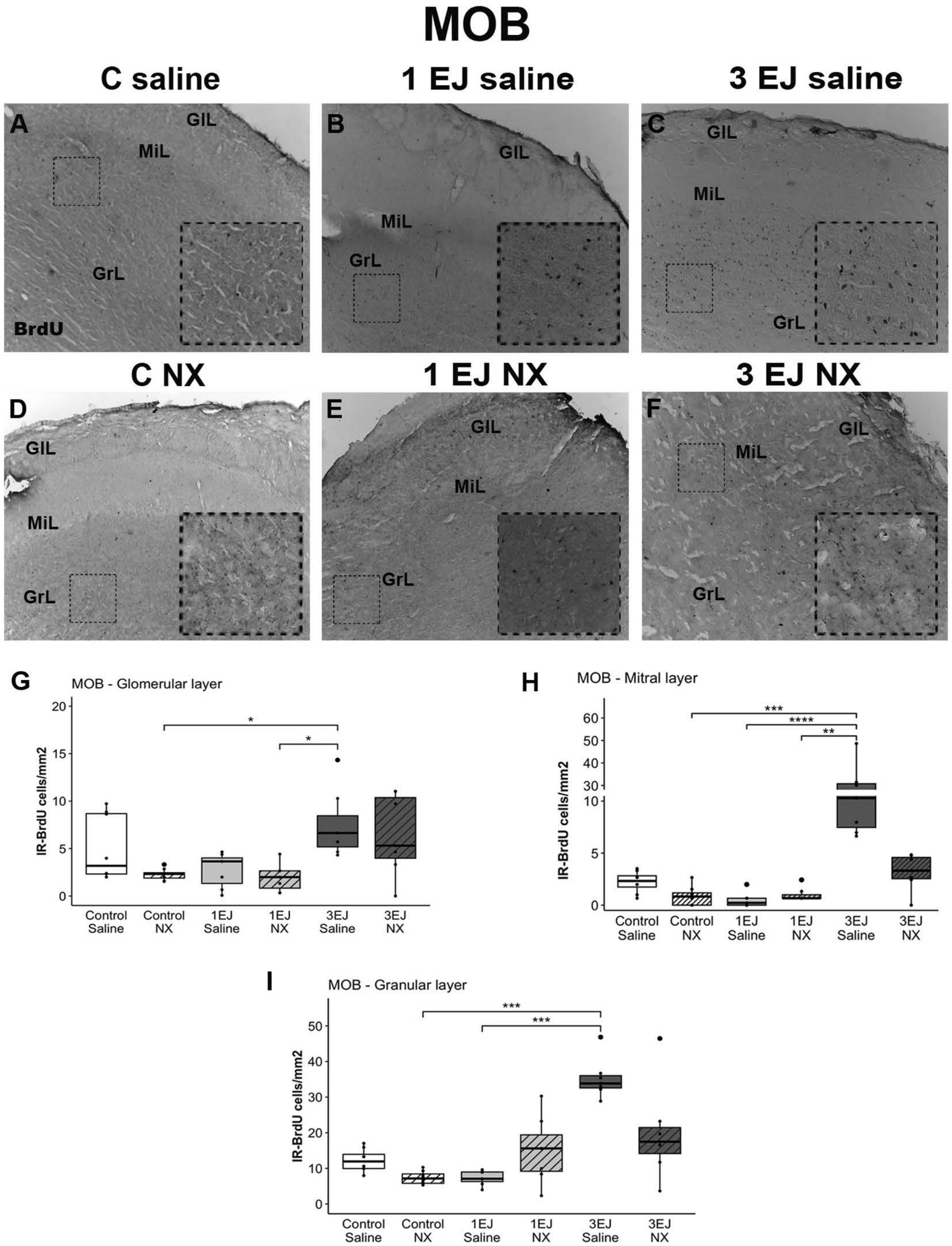

Fig 2. Effect of ejaculation and opioid blockade on cell proliferation in the main olfactory bulb (MOB). Representative photomicrographs of BrdU-positive proliferating cells (black nuclei; white arrowheads) in the MOB. (A) Control males that did not display sexual activity and were injected with saline (C saline). (B) Males that ejaculated once and were injected with saline (1 EJ saline). (C) Males that ejaculated three times and were injected with saline (3 EJ saline). (D) Control males without sexual experience and were injected with naloxone hydrochloride (C NX). (E) Males that ejaculated once and were injected with naloxone hydrochloride (1 EJ NX). (F) Males that ejaculated three times and were injected with naloxone hydrochloride (3 EJ NX). BrdU-immunoreactive (BrdU-IR) cells were identified in the glomerular cell layer (GlL), mitral layer (Mi), and granular cell layer (GrL). The insets show magnifications of the granular cell layer, highlighting (white arrowheads) proliferating cells in the experimental groups. Scale bar: 200 μm. (G, H, I) Quantification of BrdU-IR cell numbers in the GlL, Mi, and GrL, respectively, in the MOB of experimental males. * $p < 0.05$, ** $p < 0.01$, ***, $p < 0.001$, ****, $p < 0.0001$.

https://doi.org/10.1371/journal.pone.0329795.g002

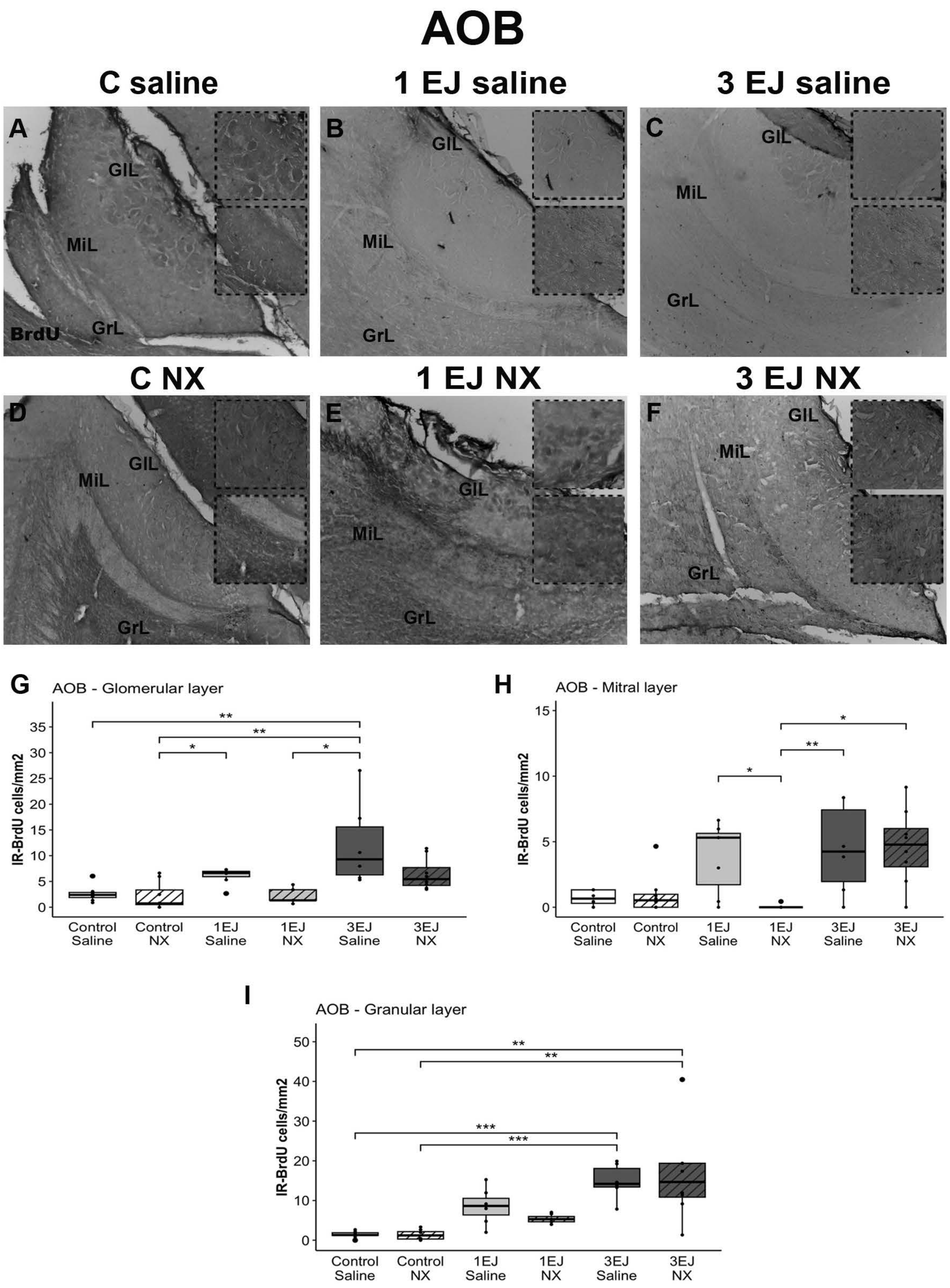

Fig 3. Effect of ejaculation and opioid blockade on cell proliferation in the accessory olfactory bulb (AOB). Representative photomicrographs of the BrdU-IR proliferating cells (black nuclei; white arrowheads) in the AOB. (A) Control males that did not display sexual activity and were injected with saline (C saline). (B) Males that ejaculated once and were injected with saline (1 EJ saline). (C) Males that ejaculated three times and were injected with saline (3 EJ saline). (D) Control males without sexual experience and were injected with naloxone hydrochloride (C NX). (E) Males that ejaculated once and were injected with naloxone hydrochloride (1 EJ NX). (F) Males that ejaculated three times and were injected with naloxone hydrochloride (3 EJ NX). BrdU-immunoreactive (BrdU-IR) cells were identified in the glomerular cell layer (GlL), mitral layer (Mi), and granular cell layer (GrL). The insets show magnifications of the granular cell layer, highlighting (white arrowheads) proliferating cells in the experimental groups. Scale bar: 200 μm. (G, H, I) Quantification of BrdU-IR cell numbers in the GlL, Mi, and GrL, respectively, in the AOB of experimental males. * $p < 0.05$, ** $p < 0.01$, ***, $p < 0.001$.

https://doi.org/10.1371/journal.pone.0329795.g003

p < 0.05 and U = −4.00, p < 0.001, respectively). Males that ejaculated 3 times and were injected with saline also showed more cell proliferation than males that were injected with NX and ejaculated either once or 3 times (U = 2.91, p < 0.05 and U = 4.20, p < 0.001, respectively). Additionally, the 1 EJ saline group had more BrdU-IR cells than the control NX and 3 EJ NX groups (U = −3.15, p < 0.05 and U = −3.37, p < 0.01, respectively). The groups treated with NX did not show a significant increase in cell proliferation (Fig 4).

In the preoptic area, Kruskal-Wallis test revealed differences between groups (H(5)=23.52, p < 0.001). The 3 EJ saline group had more BrdU-IR cells than the control groups, independently if they were injected with saline or NX (U = −3.00, p < 0.05 and U = −4.38, p < 0.001, respectively). In this brain region, males that ejaculated 3 times and were injected with saline also showed more cell proliferation than males that were injected with naloxone hydrochloride and ejaculated either once or 3 times (U = 3.72, p < 0.01 and U = 2.89, p < 0.05, respectively). The group 3 ejaculations treated with NX did not show a significant increase in cell proliferation (Fig 5).

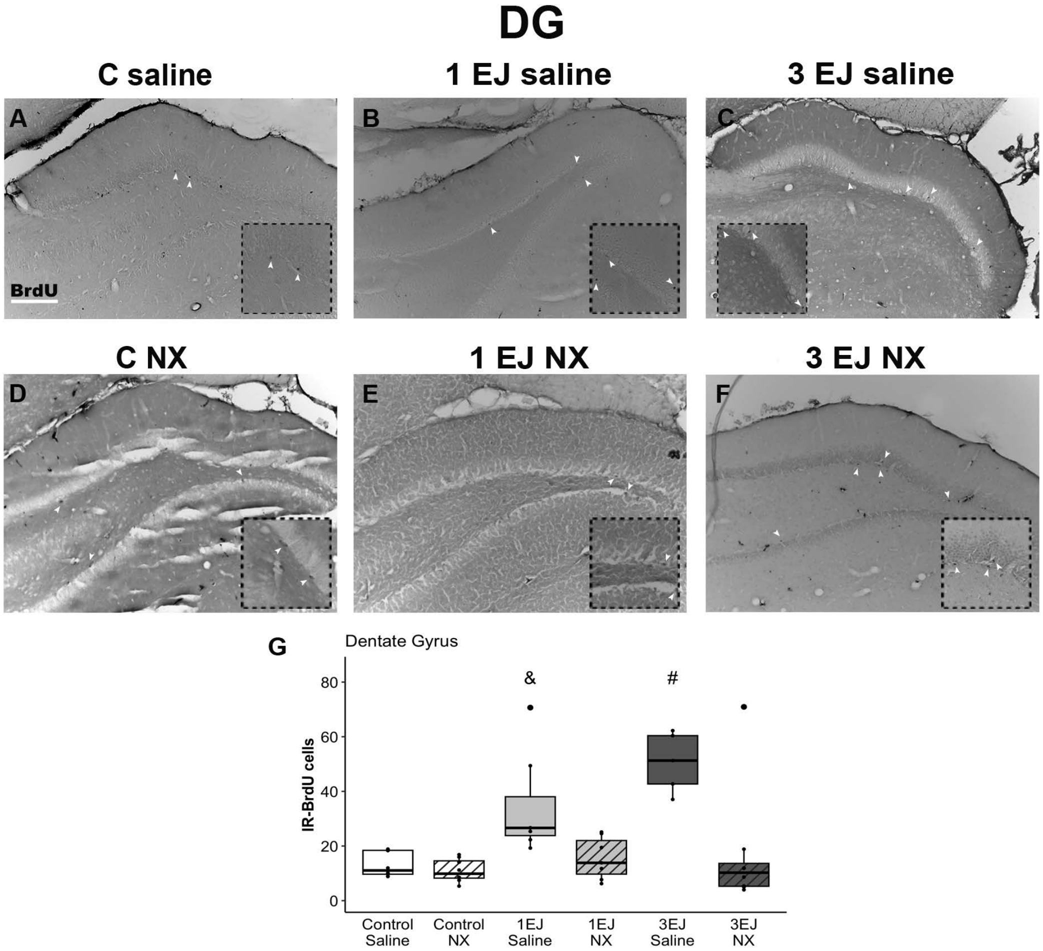

Fig 4. Effect of ejaculation and opioid blockade on cell proliferation in the dentate gyrus (DG) granular cell layer of the hippocampus. Representative photomicrographs of the BrdU-IR proliferating cells (black nuclei; white arrowheads) in the DG. (A) Control males that did not display sexual activity and were injected with saline (C saline). (B) Males that ejaculated once and were injected with saline (1 EJ saline). (C) Males that ejaculated three times and were injected with saline (3 EJ saline). (D) Control males without sexual experience and were injected with naloxone hydrochloride (C NX). (E) Males that ejaculated once and were injected with naloxone hydrochloride (1 EJ NX). (F) Males that ejaculated three times and were injected with naloxone hydrochloride (3 EJ NX). The insets show magnifications of the DG granular cell layer, highlighting (white arrowheads) proliferating cells in the experimental groups. Scale bar: 200 µm. (G) Quantification of BrdU-IR cell numbers in the DG granular cell layer. # Different from: 1EJ NX p < 0.05, 3EJ NX p < 0.001, Control Saline p < 0.05, Control NX p < 0.001; & Different from: 3EJ NX p < 0.01, Control NX p < 0.05.

https://doi.org/10.1371/journal.pone.0329795.g004

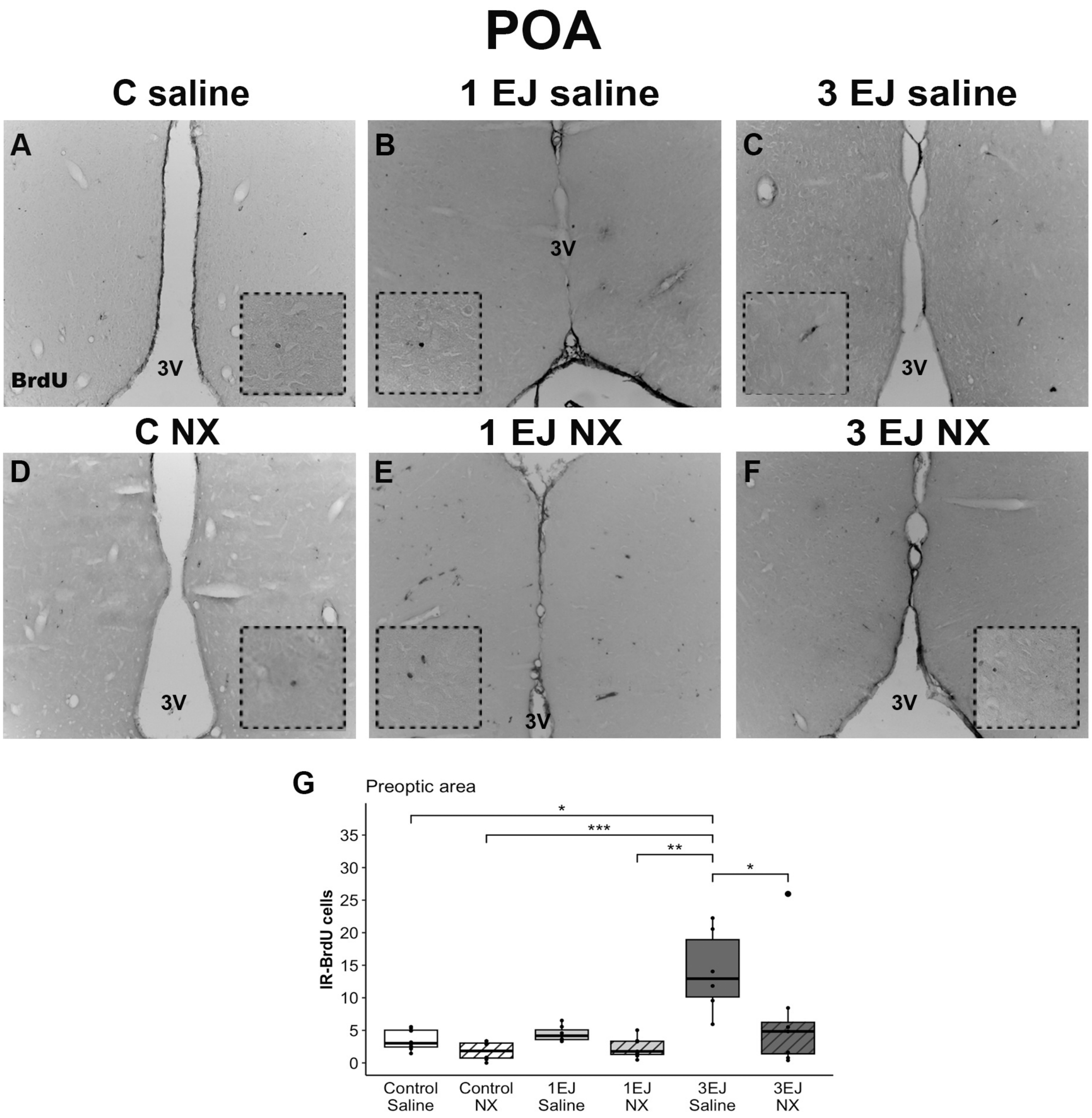

Fig 5. Effect of ejaculation and opioid blockade on cell proliferation in the preoptic area (POA). Representative photomicrographs of the BrdU-IR proliferating cells (black nuclei; white arrowheads) in the POA. (A) Control males that did not display sexual activity and were injected with saline (C saline). (B) Males that ejaculated once and were injected with saline (1 EJ saline). (C) Males that ejaculated three times and were injected with saline (3 EJ saline). (D) Control males without sexual experience and were injected with naloxone hydrochloride (C NX). (E) Males that ejaculated once and were injected with naloxone hydrochloride (1 EJ NX). (F) Males that ejaculated three times and were injected with naloxone hydrochloride (3 EJ NX). The insets show magnifications of the POA highlighting (white arrowheads) proliferating cells in the experimental groups. 3V: third ventricle. Scale bar: 200 µm. (G) Quantification of BrdU-IR cell numbers in the POA. * $p < 0.05$, ** $p < 0.01$, ***, $p < 0.001$.

https://doi.org/10.1371/journal.pone.0329795.g005

In the amygdala, Kruskal-Wallis test revealed differences between groups ($H(5) = 18.77$, $p < 0.01$). Males that ejaculated 3 times and were injected with saline also showed more cell proliferation than males that were injected with naloxone hydrochloride and ejaculated either once or 3 times ($U = 3.02$, $p < 0.05$ and $U = 3.94$, $p < 0.01$, respectively). Again, the group 3 ejaculations treated with NX did not show a significant increase in cell proliferation (Fig 6).

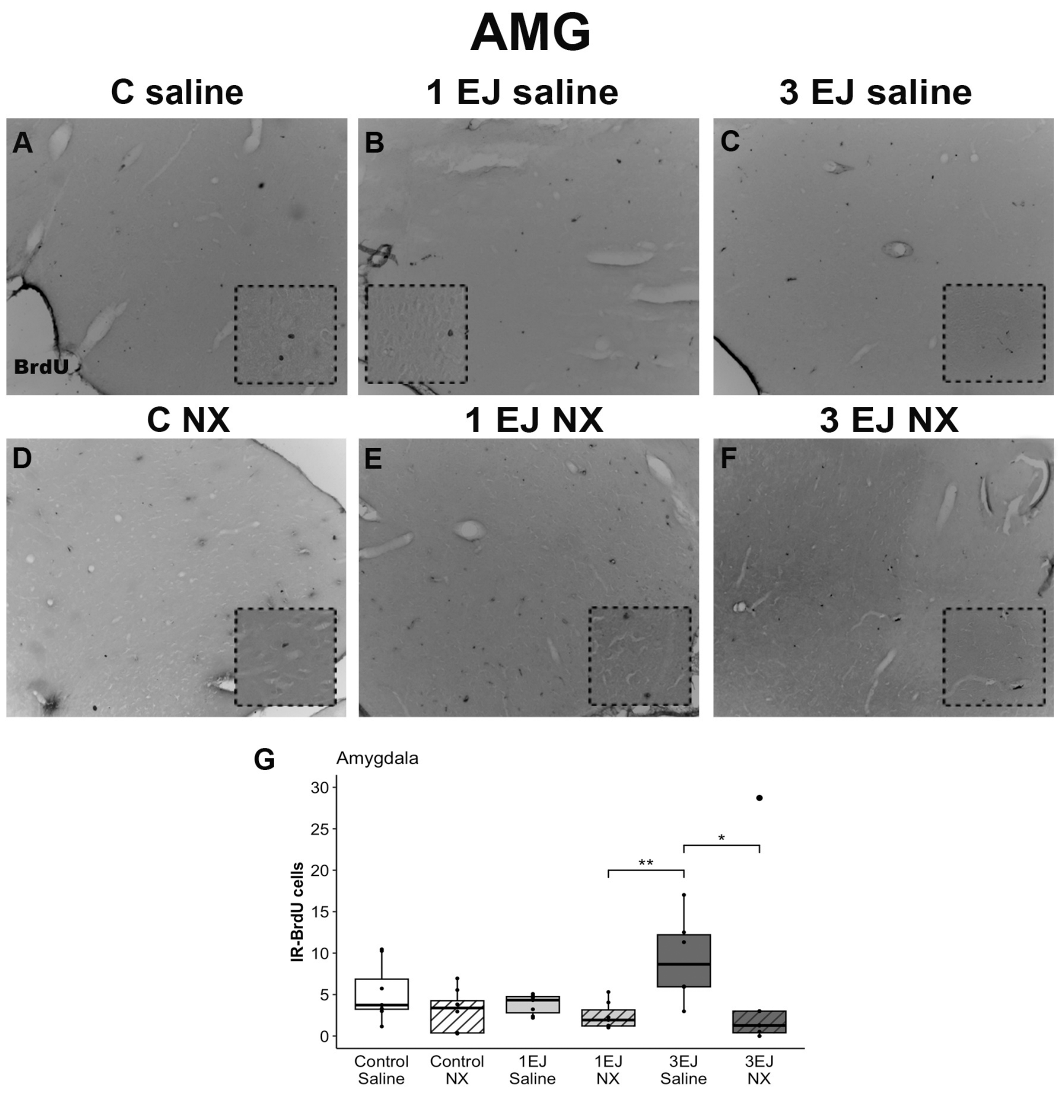

Fig 6. Effect of ejaculation and opioid blockade on cell proliferation in the amygdala (AMG). Representative photomicrographs of the BrdU-IR proliferating cells (black nuclei; white arrowheads) in the AMG. (A) Control males that did not display sexual activity and were injected with saline (C saline). (B) Males that ejaculated once and were injected with saline (1 EJ saline). (C) Males that ejaculated three times and were injected with saline (3 EJ saline). (D) Control males without sexual experience and were injected with naloxone hydrochloride (C NX). (E) Males that ejaculated once and were injected with naloxone hydrochloride (1 EJ NX). (F) Males that ejaculated three times and were injected with naloxone hydrochloride (3 EJ NX). The insets show magnifications of the AMG highlighting (white arrowheads) proliferating cells in the experimental groups. Scale bar: 200 μm. (G) Quantification of BrdU-IR cell numbers in the AMG. * $p < 0.05$, ** $p < 0.01$.

https://doi.org/10.1371/journal.pone.0329795.g006

Effect of naloxone hydrochloride and number of ejaculations on neurogenesis

Kruskal-Wallis test revealed differences between groups in the glomerular and granular layers of the MOB ($H(5)=19.47$, $p < 0.01$ and $H(5)=21.01$, $p < 0.001$, respectively). In both layers, the 3 ejaculations + saline group had more BrdU/NeuN-IR cells than control groups, independently if they were injected with saline or naloxone hydrochloride (glomerular: $U = -3.17$, $p < 0.05$ and $U = -3.78$, $p < 0.01$, respectively; granular: $U = -3.59$, $p < 0.01$ and $U = -3.59$, $p < 0.01$, respectively). The administration of NX in the 3 EJ group completely blocked the increase in neurogenesis as expressed in the number of BrdU/NeuN-IR cells (Fig 7).

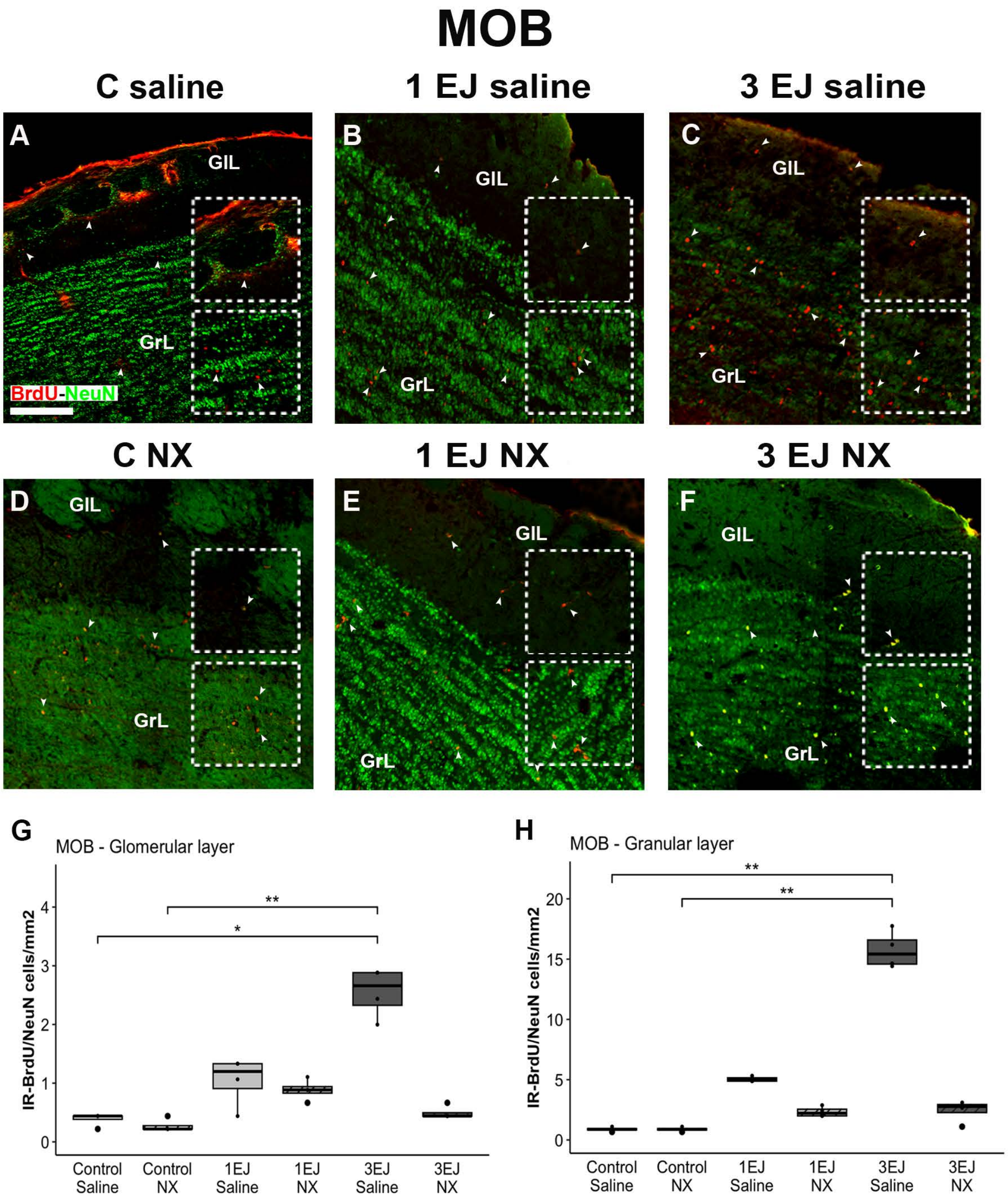

Fig 7. Effect of ejaculation and opioid blockade on neurogenesis in the main olfactory bulb (MOB). Representative photomicrographs of the double-immunolabeling for BrdU/NeuN-IR new neurons (yellow nuclei; white arrowheads) in the MOB. (A) Control males that did not display sexual activity and were injected with saline (C saline). (B) Males that ejaculated once and were injected with saline (1 EJ saline). (C) Males that ejaculated three times and were injected with saline (3 EJ saline). (D) Control males without sexual experience and were injected with hydrochloride (C NX). (E) Males that ejaculated once and were injected with naloxone hydrochloride (1 EJ NX). (F) Males that ejaculated three times and were injected with naloxone hydrochloride (3 EJ NX). Red nuclei: BrdU-positive cells. Green nuclei: NeuN-positive cells. The upper and lower insets show magnifications of the glomerular (GIL) and granular (GrL) cell layers respectively, highlighting (white arrowheads) proliferating cells in the experimental groups. Scale bar: 200 µm. (G, H) Quantification of BrdU/NeuN-IR cell numbers in the GIL and GrL of the MOB, respectively. * $p < 0.05$, ** $p < 0.01$.

https://doi.org/10.1371/journal.pone.0329795.g007

Kruskal-Wallis test revealed differences between groups in the glomerular and granular layers of the AOB ($H(5)=15.15$, $p<0.01$ and $H(5)=20.35$, $p<0.01$, respectively). In both layers, 3 ejaculations induced more neurogenesis compared to control in males that were injected with saline ($U=-3.42$, $p<0.01$ and $U=-3.43$, $p<0.01$, respectively). Additionally, in the granular layer, the 3 ejaculations + saline group had more BrdU/NeuN-IR cells than the control NX and 1 EJ NX groups ($U=-3.03$, $p<0.05$ and $U=3.23$, $p<0.05$, respectively). The administration of NX in the 3 EJ group completely blocked the increase in neurogenesis as expressed in the number of BrdU/NeuN-IR cells (Fig 8).

In the hippocampus, Kruskal-Wallis test revealed differences between groups ($H(5)=20.48$, $p<0.01$). In this brain region, the 3 ejaculations + saline group showed more neurogenesis than the control NX and 3 EJ NX groups ($U=-3.53$, $p<0.01$ and $U=3.64$, $p<0.01$). Again, the administration of NX in the 3 EJ group completely blocked the increase in neurogenesis as expressed in the number of BrdU/NeuN-IR cells (Fig 9).

Discussion

The present study aimed to investigate whether opioid signaling contributes to the observed increases in cell proliferation and neurogenesis following one or three ejaculations in male rats. We examined the olfactory bulb and dentate gyrus, the most extensively studied neurogenic niches, while also exploring the amygdala and preoptic area to provide a broader understanding of potential neurogenic activity. Globally, our findings indicate that opioid signaling plays a crucial role in cell proliferation and neurogenesis within the glomerular layer of both the AOB and MOB. In these regions, the increase of cell proliferation and neurogenesis observed after 3 ejaculations was abolished by naloxone hydrochloride administration. The same effect was observed in the granular layer of the MOB. In contrast, naloxone hydrochloride did not reduce the cell proliferation induced by 3 ejaculations in the granular layer of the AOB, but significantly reduced neurogenesis. In the dentate gyrus, cell proliferation and neurogenesis were increased by 3 ejaculations and this effect was blocked by naloxone hydrochloride. Finally, analyses of the amygdala and preoptic area revealed that naloxone hydrochloride blocked the cell proliferation induced by 3 ejaculations (Table 2).

In the present study, we found that 3 ejaculations increased cell proliferation and neurogenesis in the granular cell layer of the AOB. These results are consistent with a previous study that used the same protocol with male rats that were sacrificed 15 days after the sexual behavior interaction and the BrdU injection [26]. Interestingly, it was demonstrated that this increase persists 45 days after the sexual behavior and BrdU injection [27]. Similarly, we found that 3 ejaculations also enhanced cell proliferation and neurogenesis in the glomerular cell layer. These results contrast with previous studies that did not find any similar effect after either 15 or 45 days of survival [26,27]. In the mitral cell layer, cell proliferation and neurogenesis were not modified by sexual behavior when comparing groups with 1 or 3 ejaculations with controls. This finding is in line with what was previously shown in other studies [26,27]. The effect of naloxone hydrochloride varied depending on the cell layer. In the glomerular cell layer, the opioid antagonist reduced cell proliferation to levels comparable to the controls. However, in the granular layer, it failed to block the increase in cell proliferation induced by three ejaculations. These findings differ from those reported by Santoyo-Zedillo et al. (2017) in female rats that paced mating [18]. In their study, naloxone hydrochloride significantly reduced the number of BrdU-IR-positive cells in females allowed to pace mating for 1 hour and sacrificed 15 days later, compared to females that copulated under the same conditions but received saline injections. This difference could be due to the high variance observed in the 3EJ NX group, particularly one subject that showed a very high number of new cells. Nevertheless, naloxone hydrochloride did reduce neurogenesis in this cell layer, aligning with the findings of Santoyo-Zedillo et al. (2017) in female rats [18]. Therefore, our findings confirm that opioids contribute to the enhancement of neurogenesis induced by sexual behavior in the AOB.

To our knowledge, this study is the first to demonstrate increased cell proliferation and neurogenesis in the glomerular cell layer of the MOB in male rats following three ejaculations. Notably, these effects were effectively blocked by peripheral administration of naloxone hydrochloride. Moreover, analysis of BrdU-IR-positive cells revealed that three ejaculations also enhanced cell proliferation in the mitral and granular cell layers, an increase that was similarly inhibited by naloxone

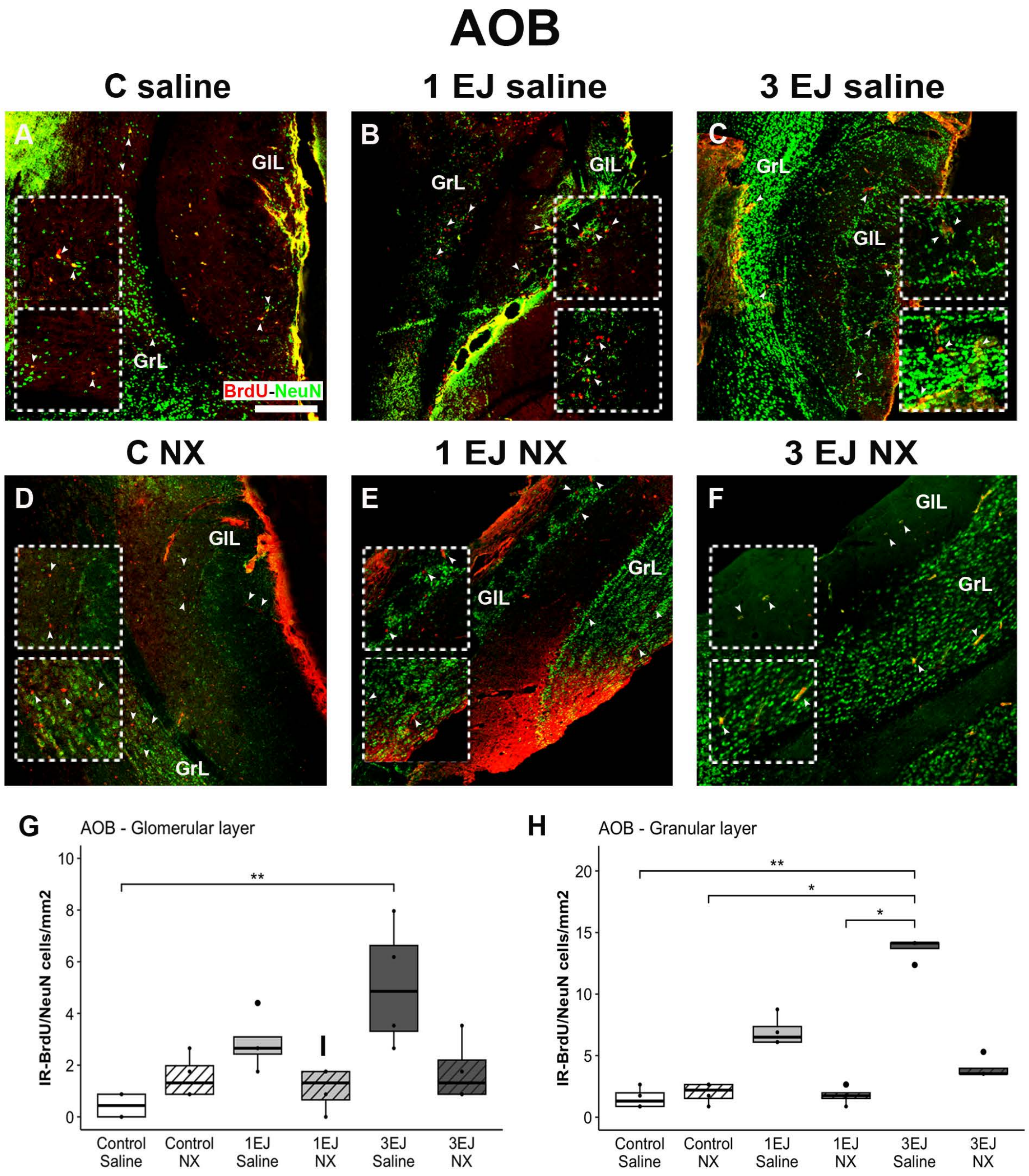

Fig 8. Effect of ejaculation and opioid blockade on neurogenesis in the accessory olfactory bulb (AOB). Representative photomicrographs of the double-immunolabeling for BrdU/NeuN-IR new neurons (yellow nuclei; white arrowheads) in the AOB. (A) Control males that did not display sexual activity and were injected with saline (C saline). (B) Males that ejaculated once and were injected with saline (1 EJ saline). (C) Males that ejaculated three times and were injected with saline (3 EJ saline). (D) Control males without sexual experience and were injected with hydrochloride (C NX). (E) Males that ejaculated once and were injected with naloxone hydrochloride (1 EJ NX). (F) Males that ejaculated three times and were injected with naloxone hydrochloride (3 EJ NX). Red nuclei: BrdU-positive cells. Green nuclei: NeuN-positive cells. The upper and lower insets show magnifications of the glomerular (GlL) and granular (GrL) cell layers respectively, highlighting (white arrowheads) proliferating cells in the experimental groups. Scale bar: 200 μm. (G, H) Quantification of BrdU/NeuN-IR cell numbers in the GlL and GrL of the AOB. * $p < 0.05$, ** $p < 0.01$.

https://doi.org/10.1371/journal.pone.0329795.g008

hydrochloride injection. Taken together, our findings demonstrate that three ejaculations significantly enhanced cell proliferation and neurogenesis across most layers of both the AOB and MOB, and this effect critically depends on opioid signaling.

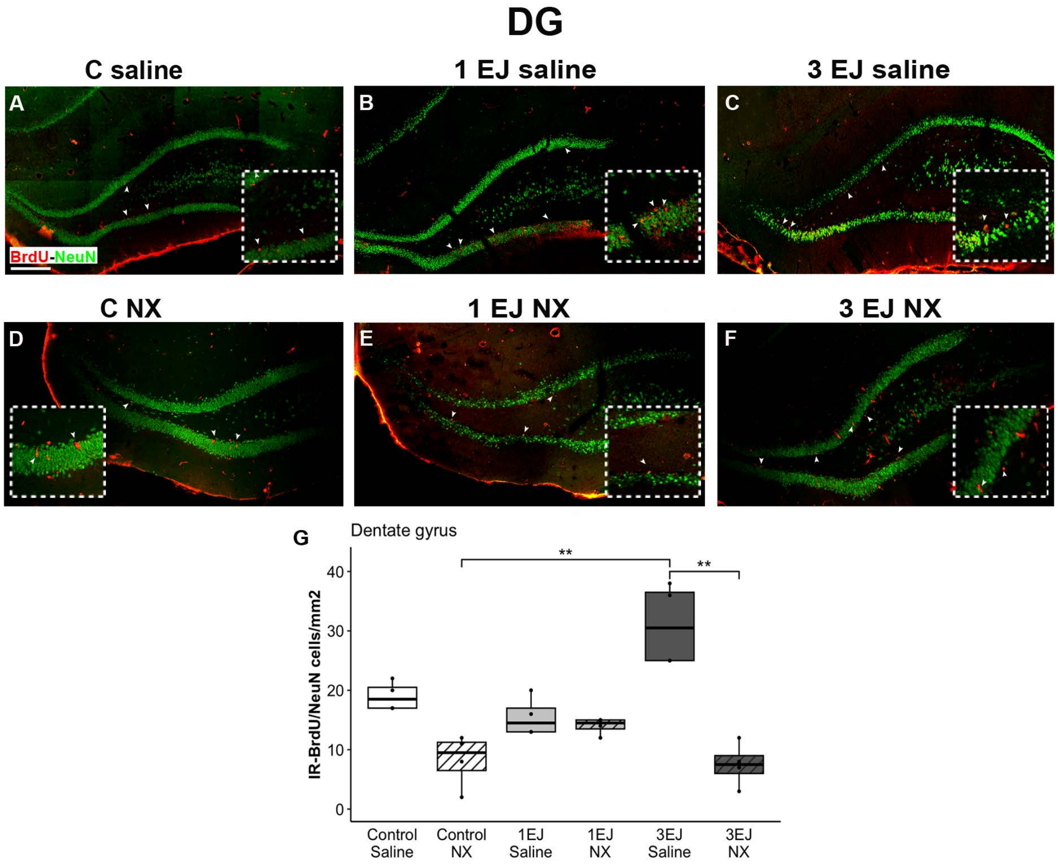

Fig 9. Effect of ejaculation and opioid blockade on neurogenesis in the dentate gyrus (DG) of the hippocampus. Representative photomicrographs of the double-immunolabeling for BrdU/NeuN-IR new neurons (yellow nuclei; white arrowheads) in the DG. (A) Control males that did not display sexual activity and were injected with saline (C saline). (B) Males that ejaculated once and were injected with saline (1 EJ saline). (C) Males that ejaculated three times and were injected with saline (3 EJ saline). (D) Control males without sexual experience and were injected with naloxone hydrochloride (C NX). (E) Males that ejaculated once and were injected with naloxone hydrochloride (1 EJ NX). (F) Males that ejaculated three times and were injected with naloxone hydrochloride (3 EJ NX). Red nuclei: BrdU-positive cells. Green nuclei: NeuN-positive cells. The inset shows a magnification of the DG granular cell layer, highlighting (white arrowheads) proliferating cells in the experimental groups. Scale bar: 200 μm. G: Quantification of BrdU/NeuN-IR cell numbers in in the DG granular cell layer. ** $p < 0.01$.

https://doi.org/10.1371/journal.pone.0329795.g009

In the dentate gyrus of the hippocampus, our study revealed that both single and three ejaculations enhanced cell proliferation. These findings align with prior research investigating the effects of acute and chronic sexual experiences. For instance, Leuner and colleagues demonstrated that a single sexual encounter or daily sexual activity over 15 days increased cell proliferation in the dentate gyrus of male rats [20]. Similarly, Glasper and Gould observed that acute and chronic sexual experiences increased cell proliferation in middle-aged male rats, a group typically exhibiting reduced hippocampal neurogenesis compared to younger counterparts [19]. In contrast, another study reported no significant differences in the number of BrdU-positive cells between male rats allowed to copulate to 1–3 ejaculations across four 30-minute sessions (spaced four days apart) and control males that remained in their home cages [28]. Our study provides novel evidence showing that the enhancement of hippocampal cell proliferation by sexual behavior is mediated through opioid signaling. Males allowed to ejaculate 1–3 times, treated with naloxone hydrochloride exhibited BrdU-IR cell numbers comparable to control animals. In contrast, males injected with saline showed a marked increase in BrdU-IR-positive cells. Additionally, the analysis of new neurons, identified by BrdU and NeuN colocalization, revealed

Table 2. Summary of the principal findings.

Region	Cell layer	New cells (BrdU +)	New neurons (BrdU-NeuN +)
**MOB**	Glomerular	↑ 3 EJ Saline (vs Control Saline; Control NX; 1EJ NX)	↑ 3 EJ Saline (vs Control Saline; Control NX)
	Mitral	↑ 3 EJ Saline (Control NX; 1 EJ Saline; 1EJ NX)	ND
	Granular	↑ 3 EJ Saline (Control NX; 1 EJ Saline)	↑ 3 EJ Saline (vs Control Saline; Control NX)
**AOB**	Glomerular	↑ 1 EJ Saline (vs Control NX)	
		↑ 3 EJ Saline (vs Control Saline; Control NX; 1 EJ NX)	↑ 3 EJ Saline (vs Control Saline)
	Mitral	↓ 1 EJ NX (vs 1 EJ Saline; 3 EJ Saline; 3 EJ NX)	ND
	Granular	↑ 3 EJ Saline (vs Control Saline; Control NX)	↑ 3 EJ Saline (vs Control Saline; Control NX; 1EJ NX)
		↑ 3 EJ NX (vs Control Saline; Control NX)	
**DG**		NS	↑ 3 EJ Saline (vs Control NX; 3 EJ NX)
**POA**		↑ 3 EJ Saline (vs Control Saline; Control NX; 1 EJ NX; 3 EJ NX)	ND
**AMG**		↑ 3 EJ Saline (vs 1 EJ NX; 3 EJ NX)	ND

Effects of 1 or 3 ejaculations and naloxone hydrochloride (NX) administration on new cells/new neurons in male rats. ↑ increase ↓ decrease in the number of new cells/new neurons. ND: not determined. NS: not significant.

https://doi.org/10.1371/journal.pone.0329795.t002

that naloxone hydrochloride abolished the increase in neurogenesis observed in males that ejaculated three times. Taken together, these findings confirm that sexual behavior enhances cell proliferation and neurogenesis in the dentate gyrus of male rats. Moreover, our study demonstrates that this effect is mediated by opioid signaling.

In addition to examining the classical neurogenic niches, we investigated cell proliferation in the POA and AMG, brain regions involved in sexual behavior. Notably, we found that administering naloxone hydrochloride prior to sexual activity significantly reduced the number of newly formed cells in both the amygdala and the preoptic area. Analyses revealed that three ejaculations led to an increase in cell proliferation in both regions. However, this increase was effectively blocked by naloxone hydrochloride, as evidenced by a significant reduction in BrdU-IR-positive cells in the 3EJ NX group compared to the 3EJ Saline group. Our results contrast with what was described in male hamsters [29]. Indeed, they found no effect of acute or chronic sexual experience on the cell proliferation observed in the posterior medial amygdala or preoptic area.

Our results provide further evidence that opioids modulate cell proliferation and neurogenesis not only within the classical neurogenic niches, such as the olfactory bulb and the dentate gyrus of the hippocampus, but also in other brain regions associated with socio-sexual behaviors. Several studies have demonstrated that opioids modulate cell differentiation in the hippocampus. For instance, short-term exposure to morphine promoted neuronal and glial differentiation while reducing the number of nestin-positive cells, effects that were counteracted by the opioid antagonist naloxone hydrochloride [30]. Likewise, β-Endorphin was found to enhance proliferation in cultured rat adult hippocampal progenitors, an effect that was blocked by DOR and MOR antagonists. Agonists targeting these receptors also stimulated proliferation, highlighting the involvement of both receptor subtypes in this response [31]. In related work, the same group demonstrated that naloxone hydrochloride reduced proliferation in cultured rat adult hippocampal progenitors [32]. Together, these findings support the conclusion that opioid signaling plays a key role in regulating proliferation and neurogenesis across diverse brain regions.

Our findings reveal that the increase in cell proliferation observed in males that engaged in copulation was largely, but not entirely, prevented by naloxone hydrochloride treatment across the studied regions. This suggests that, beyond opioids, other hormones or neurotransmitters likely contribute to the effects of sexual behavior on neurogenesis. For instance, oxytocin has been shown to enhance cell proliferation in the hippocampus following both peripheral and central administration in adult male rats [33]. Additionally, the hippocampus is enriched with prolactin receptors, which may play a role in mediating the effects of sexual experience on adult neurogenesis. Supporting this, male pheromones have been demonstrated to regulate neurogenesis through prolactin signaling in both the olfactory bulb and hippocampus of female

mice [34]. Furthermore, as previously reviewed, hippocampal neurogenesis in adults is influenced by gonadal hormones in a manner dependent on both sex and experience [35,36]. Importantly, evidence suggests that gonadal hormones facilitate adult neurogenesis and serve as neuroprotective agents for adult-generated cells by interacting with BDNF (Brain Derived Neurotrophic Factor), serotonin, and oxytocin [37]. Further studies need to test if these neuromodulators play a role in cell proliferation and neurogenesis induced by mating.

Finally, our behavioral analysis confirmed that naloxone hydrochloride did not affect sexual behavior of male rats, as previously reported [17]. Copulatory parameters, including latencies, frequencies, post-ejaculatory intervals, and inter-intromission intervals, were consistent across saline- and naloxone hydrochloride-treated groups. As expected, males in the 3 EJ groups exhibited more frequent mounts and intromissions than those in the one-ejaculation groups, regardless of treatment, reflecting the greater opportunity for multiple ejaculations.

In conclusion, this study highlights the central role of opioid signaling in mediating the effects of sexual behavior on cell proliferation and neurogenesis in both classical and non-classical neurogenic regions. These findings advance our understanding of the neurobiological mechanisms underlying the interaction between sexual behavior and adult neurogenesis and open avenues for exploring the contributions of other signaling systems in this context.

Supporting information

S1 File. Data set. Behavioral data and individual values from the quantitative analysis of BrdU-IR and BrdU/NeuN-IR cells.
(XLSX)

Acknowledgments

We thank Francisco Camacho, Martín García, and Sandra Hernández for their excellent technical assistance. We also appreciate the support of Alejandra Castilla León and María A. Carbajo in providing the experimental subjects.

Author contributions

Conceptualization: Raúl G. Paredes.

Data curation: Mariana Sánchez Núñez, Analia E. Castro.

Formal analysis: Marie Bedos.

Funding acquisition: Marie Bedos, Raúl G. Paredes.

Investigation: Mariana Sánchez Núñez, Analia E. Castro.

Project administration: Raúl G. Paredes.

Supervision: Raúl G. Paredes.

Visualization: Marie Bedos.

Writing – original draft: Marie Bedos.

Writing – review & editing: Analia E. Castro, Raúl G. Paredes.

References

1. Leal-Galicia P, Chávez-Hernández ME, Mata F, Mata-Luévanos J, Rodríguez-Serrano LM, Tapia-de-Jesús A, et al. Adult Neurogenesis: A Story Ranging from Controversial New Neurogenic Areas and Human Adult Neurogenesis to Molecular Regulation. Int J Mol Sci. 2021;22(21):11489. https://doi.org/10.3390/ijms222111489 PMID: 34768919
2. Portillo W, Nuñez M, Paredes RG, Bedos M. Neurobiology of Reproductive Behavior, The Role of Neurogenesis. Encyclopedia of Animal Behavior. Elsevier. 2019. p. 274–82. https://doi.org/10.1016/b978-0-12-809633-8.90713-1

3. Lledo P-M, Alonso M, Grubb MS. Adult neurogenesis and functional plasticity in neuronal circuits. Nat Rev Neurosci. 2006;7(3):179–93. https://doi.org/10.1038/nrn1867 PMID: 16495940
4. Lazarov O, Mattson MP, Peterson DA, Pimplikar SW, van Praag H. When neurogenesis encounters aging and disease. Trends in Neurosciences. 2010;33(12):569–79. https://doi.org/10.1016/j.tins.2010.09.003
5. Ming G-L, Song H. Adult neurogenesis in the mammalian brain: significant answers and significant questions. Neuron. 2011;70(4):687–702. https://doi.org/10.1016/j.neuron.2011.05.001 PMID: 21609825
6. Denoth-Lippuner A, Jessberger S. Formation and integration of new neurons in the adult hippocampus. Nat Rev Neurosci. 2021;22(4):223–36. https://doi.org/10.1038/s41583-021-00433-z PMID: 33633402
7. Gage FH. Adult neurogenesis in mammals. Science. 2019;364(6443):827–8. https://doi.org/10.1126/science.aav6885 PMID: 31147506
8. Camacho F, Sandoval C, Paredes RG. Sexual experience and conditioned place preference in male rats. Pharmacol Biochem Behav. 2004;78(3):419–25. https://doi.org/10.1016/j.pbb.2004.04.015 PMID: 15251250
9. Camacho FJ, García-Horsman P, Paredes RG. Hormonal and testing conditions for the induction of conditioned place preference by paced mating. Horm Behav. 2009;56(4):410–5. https://doi.org/10.1016/j.yhbeh.2009.07.007 PMID: 19646448
10. Corona R, Camacho FJ, García-Horsman P, Guerrero A, Ogando A, Paredes RG. Different doses of estradiol benzoate induce conditioned place preference after paced mating. Horm Behav. 2011;60(3):264–8. https://doi.org/10.1016/j.yhbeh.2011.05.013 PMID: 21712041
11. Martínez I, Paredes RG. Only self-paced mating is rewarding in rats of both sexes. Horm Behav. 2001;40(4):510–7. https://doi.org/10.1006/hbeh.2001.1712 PMID: 11716580
12. Paredes RG, Martínez I. Naloxone blocks place preference conditioning after paced mating in female rats. Behav Neurosci. 2001;115(6):1363–7. https://doi.org/10.1037//0735-7044.115.6.1363 PMID: 11770067
13. García-Horsman SP, Agmo A, Paredes RG. Infusions of naloxone into the medial preoptic area, ventromedial nucleus of the hypothalamus, and amygdala block conditioned place preference induced by paced mating behavior. Horm Behav. 2008;54(5):709–16. https://doi.org/10.1016/j.yhbeh.2008.07.011 PMID: 18721808
14. Agmo A, Berenfeld R. Reinforcing properties of ejaculation in the male rat: role of opioids and dopamine. Behav Neurosci. 1990;104(1):177–82. https://doi.org/10.1037//0735-7044.104.1.177 PMID: 2156520
15. Mehrara BJ, Baum MJ. Naloxone disrupts the expression but not the acquisition by male rats of a conditioned place preference response for an oestrous female. Psychopharmacology (Berl). 1990;101(1):118–25. https://doi.org/10.1007/BF02253728 PMID: 2343072
16. Ågmo A, Gómez M. Sexual reinforcement is blocked by infusion of naloxone into the medial preoptic area. Behavioral Neuroscience. 1993;107(5):812–8. https://doi.org/10.1037/0735-7044.107.5.812
17. Agmo A, Paredes R. Opioids and sexual behavior in the male rat. Pharmacol Biochem Behav. 1988;30(4):1021–34. https://doi.org/10.1016/0091-3057(88)90135-9 PMID: 3227027
18. Santoyo-Zedillo M, Portillo W, Paredes RG. Neurogenesis in the olfactory bulb induced by paced mating in the female rat is opioid dependent. PLoS One. 2017;12(11):e0186335. https://doi.org/10.1371/journal.pone.0186335 PMID: 29107945
19. Glasper ER, Gould E. Sexual experience restores age-related decline in adult neurogenesis and hippocampal function. Hippocampus. 2013;23(4):303–12. https://doi.org/10.1002/hipo.22090 PMID: 23460298
20. Leuner B, Glasper ER, Gould E. Sexual experience promotes adult neurogenesis in the hippocampus despite an initial elevation in stress hormones. PLoS One. 2010;5(7):e11597. https://doi.org/10.1371/journal.pone.0011597 PMID: 20644737
21. Sánchez-Vidaña DI, Chan N-MJ, Chan AHL, Hui KKY, Lee S, Chan H-Y, et al. Repeated treatment with oxytocin promotes hippocampal cell proliferation, dendritic maturation and affects socio-emotional behavior. Neuroscience. 2016;333:65–77. https://doi.org/10.1016/j.neuroscience.2016.07.005 PMID: 27418343
22. Zhang Y, Loh HH, Law P-Y. Effect of Opioid on Adult Hippocampal Neurogenesis. Sci World J. 2016;2016:2601264. https://doi.org/10.1155/2016/2601264 PMID: 27127799
23. Fowler CD, Liu Y, Wang Z. Estrogen and adult neurogenesis in the amygdala and hypothalamus. Brain Res Rev. 2008;57(2):342–51. https://doi.org/10.1016/j.brainresrev.2007.06.011 PMID: 17764748
24. Fowler CD, Freeman ME, Wang Z. Newly proliferated cells in the adult male amygdala are affected by gonadal steroid hormones. J Neurobiol. 2003;57(3):257–69. https://doi.org/10.1002/neu.10273 PMID: 14608662
25. Castro AE, Domínguez-Ordoñez R, Young LJ, Camacho FJ, Ávila-González D, Paredes RG, et al. Pair-bonding and social experience modulate new neurons survival in adult male and female prairie voles (Microtus ochrogaster). Front Neuroanat. 2022;16. https://doi.org/10.3389/fnana.2022.987229
26. Portillo W, Unda N, Camacho FJ, Sánchez M, Corona R, Arzate DMa, et al. Sexual activity increases the number of newborn cells in the accessory olfactory bulb of male rats. Front Neuroanat. 2012;6. https://doi.org/10.3389/fnana.2012.00025
27. Unda NM, Portillo W, Corona R, Paredes RG. Sexual Stimulation Increases the Survival of New Cells in the Accessory Olfactory Bulb of the Male Rat. Front Neurosci. 2016;10:65. https://doi.org/10.3389/fnins.2016.00065 PMID: 26973447
28. Spritzer MD, Curtis MG, DeLoach JP, Maher J, Shulman LM. Sexual interactions with unfamiliar females reduce hippocampal neurogenesis among adult male rats. Neuroscience. 2016;318:143–56. https://doi.org/10.1016/j.neuroscience.2016.01.015 PMID: 26794592

29. Antzoulatos E, Magorien JE, Wood RI. Cell proliferation and survival in the mating circuit of adult male hamsters: effects of testosterone and sexual behavior. Horm Behav. 2008;54(5):735–40. https://doi.org/10.1016/j.yhbeh.2008.08.001 PMID: 18775431

30. Willner D, Cohen-Yeshurun A, Avidan A, Ozersky V, Shohami E, Leker RR. Short term morphine exposure in vitro alters proliferation and differentiation of neural progenitor cells and promotes apoptosis via mu receptors. PLoS One. 2014;9(7):e103043. https://doi.org/10.1371/journal.pone.0103043 PMID: 25072277

31. Persson AI, Thorlin T, Bull C, Eriksson PS. Opioid-induced proliferation through the MAPK pathway in cultures of adult hippocampal progenitors. Mol Cell Neurosci. 2003;23(3):360–72. https://doi.org/10.1016/s1044-7431(03)00061-7 PMID: 12837621

32. Persson AI, Thorlin T, Bull C, Zarnegar P, Ekman R, Terenius L, et al. Mu- and delta-opioid receptor antagonists decrease proliferation and increase neurogenesis in cultures of rat adult hippocampal progenitors. Eur J Neurosci. 2003;17(6):1159–72. https://doi.org/10.1046/j.1460-9568.2003.02538.x PMID: 12670304

33. Leuner B, Caponiti JM, Gould E. Oxytocin stimulates adult neurogenesis even under conditions of stress and elevated glucocorticoids. Hippocampus. 2012;22(4):861–8. https://doi.org/10.1002/hipo.20947 PMID: 21692136

34. Mak GK, Enwere EK, Gregg C, Pakarainen T, Poutanen M, Huhtaniemi I, et al. Male pheromone-stimulated neurogenesis in the adult female brain: possible role in mating behavior. Nat Neurosci. 2007;10(8):1003–11. https://doi.org/10.1038/nn1928 PMID: 17603480

35. Galea LAM, Wainwright SR, Roes MM, Duarte-Guterman P, Chow C, Hamson DK. Sex, hormones and neurogenesis in the hippocampus: hormonal modulation of neurogenesis and potential functional implications. J Neuroendocrinol. 2013;25(11):1039–61. https://doi.org/10.1111/jne.12070 PMID: 23822747

36. Opendak M, Briones BA, Gould E. Social behavior, hormones and adult neurogenesis. Frontiers in Neuroendocrinology. 2016;41:71–86. https://doi.org/10.1016/j.yfrne.2016.02.002

37. Jorgensen C, Wang Z. Hormonal Regulation of Mammalian Adult Neurogenesis: A Multifaceted Mechanism. Biomolecules. 2020;10(8):1151. https://doi.org/10.3390/biom10081151
